# Early diabetes screening in women with previous gestational diabetes: a new insight

**DOI:** 10.1186/s13098-016-0172-2

**Published:** 2016-08-27

**Authors:** Aline Nabuco, Samara Pimentel, Carolina A. Cabizuca, Melanie Rodacki, Denise Finamore, Marcus M. Oliveira, Lenita Zajdenverg

**Affiliations:** 1 Nutrology and Diabetes Section/Maternidade Escola, Universidade Federal do Rio de Janeiro, Rio de Janeiro, Rio de Janeiro CEP 21941-913 Brazil; 2 Maternidade Escola, Universidade Federal do Rio de Janeiro, Rio de Janeiro, Brazil; 3 Serviço de Nutrologia e Diabetes, Hospital Universitário Clementino Fraga Filho, Rua Professor Rodolpho Paulo Rocco 255, sala 9E14, University City, CEP 21941-913 Brazil

**Keywords:** Gestational diabetes mellitus, Postpartum screening, Puerperium

## Abstract

**Background:**

Gestational diabetes mellitus (GDM) is a risk factor for the development of diabetes mellitus (DM). However, there is a low return rate for this screening, so it is important to search for earlier methods for evaluation after delivery, to increase the number of pregnant women screened, so you can start the treatment or prevention of these early comorbidities. To determine the accuracy of the 75 g 2-h oral glucose tolerance test (OGTT) performed between 48–72 h after delivery for the diagnosis of DM using the OGTT after 6 weeks as the gold standard criterion, and to identify the optimal cutoff points for this exam for diabetes screening after a pregnancy complicated by GDM.

**Methods:**

82 women with previous GDM underwent an OGTT between 48–72 h postpartum and repeated the test 6 weeks after delivery.

**Results:**

The prevalence of DM and prediabetes based on the first OGTT was 3.7 and 32.9 %, respectively, and 8.5 and 20.7 %, respectively, at the second OGTT. For those with DM, the area under the curve (AUC) based on the fasting plasma glucose (FPG) was 0.77 (95 % CI 0.61–0.92), and based on 2-h OGTT was 0.82 (95 % CI 0.66–0.97). For patients with prediabetes, the AUC based on the FPG was 0.73 (95 % CI 0.59–0.86) and based on the 2-h OGTT was 0.74 (95 % CI 0.61–0.87). Using a FPG cutoff value of 78 mg/dl (4.3 mmol/L) and a 2-h OGTT cutoff value of 130 mg/dl (7.2 mmol/L) for DM, the specificity was 58.7 and 60 %, the sensitivity was 71.4 and 85.7 %, the positive predictive value was 13.9 and 16.7 and the negative predictive value was 95.7 and 97.9 %, respectively.

**Conclusions:**

OGTT performed early in postpartum is a useful tool for identifying women with previous GDM who must perform an OGTT 6 weeks after delivery.

## Background

The number of patients with diabetes mellitus (DM) has increased significantly in recent decades. Despite better awareness and developments in treatment and prevention of type 2 diabetes, one in two adults with diabetes is undiagnosed [1]. The increasing prevalence of overweight and obesity in both developed and developing countries are the main factors for this rise [2]. Likewise, a growing number of cases of gestational diabetes mellitus (GDM) have been described in the last decades [3].

The frequency of GDM varies between 3–14 % depending on the method used for diagnosis and the study population [4–7]. The magnitude of the risk of postpartum diabetes depends on the ethnicity, the duration of follow up and the specific criteria for GDM diagnosis. Studies have shown that 3–65 % of women with previous GDM develop type 2 diabetes within 5–16 years after the index pregnancy [8–13]. When screened 6–12 weeks postpartum, up to 10 % of women who had GDM were diagnosed with diabetes and an additional 12–36 % had impaired fasting glucose or impaired glucose tolerance [14, 15]. Postpartum screening aims to identify women that developed or have an elevated risk of developing diabetes after pregnancy. Early recognition is important because lifestyle modifications and medications can reduce the incidence of diabetes in individuals at high risk [16–18]. Additionally, the early treatment of diabetes can prevent or delay microvascular end organ complications and reduce the risk of experiencing complications in subsequent pregnancies [19–23].

Both the American Diabetes Association (ADA) and the World Health Organisation (WHO) recommend postpartum screening after 6–12 weeks, using the 75 g 2-h oral glucose tolerance test (OGTT) [24–26]. The United Kingdom’s National Institute for Health and Clinical Excellence (NICE) recommends the fasting plasma glucose (FPG) test be administered at least 6 weeks after childbirth, instead of the traditional OGTT [27, 28]. The OGTT is more sensitive, with reported sensitivities of 100 % compared with 67 % for the FPG [29]. Previous studies of postpartum diabetes screening in women with GDM-affected pregnancies have noted test completion rates that range from 14–61 % [15, 20, 30, 31]. Alternative diagnostic tools may increase the number of evaluated women with previous GDM. The ADA recommends that women with a history of GDM with a normal postpartum screening might be rescreened every 3 years, and women with impaired fasting glucose (IFG) or impaired glucose tolerance (IGT) or both (prediabetes) should be rescreened annually [18].

The purpose of this study was to determine the accuracy of the 2-h OGTT performed between 48–72 h after delivery for the diagnosis of diabetes and to identify the optimal 2-h OGTT cutoff points for screening dysglycaemia in the early postpartum period using the follow-up 2-h OGTT after 6 weeks as the gold standard criteria.

## Methods

### Study design

In this prospective observational study, women with previous GDM who were recruited from a multi-ethnic population were evaluated. The diagnosis of GDM in pregnant women prior to December 2010 was made according to the Carpenter and Coustan criteria [32]; after January 2011, the diagnosis was made according to the International Association of Diabetes and Pregnancy Study Groups (IADPSG) [33]. The inclusion criteria were pregnant women diagnosed with GDM with regular follow-up in the diabetes and pregnancy outpatient clinic of the maternity school at Rio de Janeiro Federal University. Women who used medications known to affect glucose metabolism, and mothers diagnosed with GDM who were discharged before 48 h after delivery were excluded (8.1 %). All women identified with GDM underwent self-monitoring of blood glucose measurements, as well as dietary management. Insulin treatment was initiated when dietary management did not achieve the glycaemic goal (fasting blood glucose >95 mg/dl, 1 h postprandial blood glucose >140 mg/dl, or 2 h postprandial blood glucose >120 mg/dl). The standard care was to screen all pregnant women with previous GDM with the 2-h OGTT at six weeks after delivery.

Data collection included a detailed clinical and obstetric history. Measurements of FPG and a 2-h OGTT were assessed using an enzymatic colorimetric method between 48–72 h and 6 weeks after delivery. Study subjects were instructed to fast overnight for at least 8 h prior to their testing day and to eat at least 150 g of carbohydrate the day prior to testing. The OGTT used a 75 g anhydrous glucose load and followed the standard WHO procedures [34]. The diagnostic categories of normal, prediabetes (i.e., impaired fasting glucose or impaired glucose tolerance) and diabetes were determined from the results of the FPG and 2-h OGTT using the WHO 2006/ADA criteria [35].

### Ethical considerations

All participants provided written informed consent. The Local Ethics Committee approved this study.

### Statistical analysis

Statistical analyses were performed with SPSS version 20.0. Differences in the classifications between normal, prediabetes and diabetes using the FPG and OGTT were assessed using a non-parametric test (Wilcoxon). Receiver operating characteristic (ROC) curves were developed, and the area under the curve (AUC) with 95 % CIs was determined. The ROC curves were constructed to calculate the sensitivity, specificity, predictive value positive and predictive value negative at different cutoff values. The optimal FPG and 2 h OGTT-screening cutoff points between 48–72 h after delivery were determined by taking the greatest sum of the sensitivity and specificity for the measured FPG and 2-h OGTT values between the two diagnosed groups (diabetes and prediabetes). The positive predictive value was defined as the number of true positives divided by the total number of individuals who tested positive, whereas the negative predictive value referred to the proportion of subjects with a negative test result who were correctly diagnosed. The positive predictive value and negative predictive value were also reported for the optimal cutoff values.

## Results and discussion

During the inclusion period, 257 women with GDM were identified; however, only 82 (31.9 %) patients met all inclusion criteria, had full laboratory data for analysis, and were included in the study. In fact, 21 mothers were discharged before 48 h (8.1 %), 49 patients did only the first OGTT (19 %), and 105 did not return to the second OGTT (40, 85 %). Perinatal features of this study cohort were as follows: the mean age was 32.2 (± 5.8) years, the mean body mass index (BMI) was = 27.7 (±5.3) kg/m^2^, 54.4 % were non-caucasian, 56.7 % had more than 8 years of education and 68.4 % had a relative with DM. Additionally, the mean parity of the women was 2.3 (±1.3), the mean gestational age at GDM diagnosis was 23.1 weeks (±7.4), 69.4 % had a caesarean delivery and 64.6 % required insulin treatment (Table 1).The mean FPG levels in the early period (48–72 h) after delivery were 76.7 mg/dl (4.26 mmol/L ± 0.66), whereas the mean FPG levels after 6 weeks were 92.6 mg/dl (5.1 mmol/L ± 15.7) (p < 0.0001). All patients were breastfeeding during routine postpartum OGTT. The 2 h post-load glucose was 123.6 mg/dl (6.8 mmol/L ± 2.0) and 110.0 mg/dl (6.1 mmol/L (±2.2) (p = 0.001) between 48–72 h and after 6 weeks, respectively. The prevalence of diabetes and prediabetes based on the 75 g OGTT performed at 48–72 h after birth was 3.7 and 32.9 %, respectively, whereas the prevalence based on the second OGTT was 8.5 and 20.7 %, respectively.

**Table 1:** Patient characteristics

Characteristics	Mean ± SD or %
Age (years)	32.2 ± 5.8
Gestational age at diagnosis (weeks)	23.1 ± 7.4
Parity (n)	2.3 ± 1.3
Weight gain until delivery (kg)	10.2 ± 6.3
Pre-pregnancy BMI (kg/m^2^)	27.7 ± 5.3
Gestational age at birth (weeks)	37.8 ± 2.9

Women with diagnosis of diabetes on the second 75 g OGTT (n = 7) had mean age of 33.1 (±6.4) years and the mean BMI was 29.8 (±7.2) kg/m^2^ respectively. In addition, the mean parity was 2.4 (±1.6), and the mean gestational age at GDM diagnosis was 19.4 (±8.7). These patients had a mean weight gain until the GDM diagnosis of 5.1 (±3.8) kg and 8.2 (±2.9) kg until delivery. Eighty-five percent were on using insulin at an average of 27.3 (±2.9) weeks, and seventy-one percent had a family history of DM. The delivery was on average 37.5 (±5.2) weeks and the neonate’s weight was 3108.3 (±957.2) g. There was no statistically significant difference between mothers with and without a diagnosis of diabetes postpartum.

Using the 2-h value of 200 mg/dl (11.1 mmol/L) as the cutoff for identifying individuals with diabetes resulted in a sensitivity of 28.6 %, a specificity of 98.7 % and positive and negative predictive values of 66.7 and 93.7 %, respectively. The performance of the early 2-h OGTT was also evaluated using 140 mg/dl (7.7 mmol/L) as the cutoff for identifying individuals with prediabetes, which revealed a sensitivity of 64.7 %, a specificity of 75.4 % and positive and negative predictive values of 40.7 and 89.1 %, respectively.

**Fig. 1:**
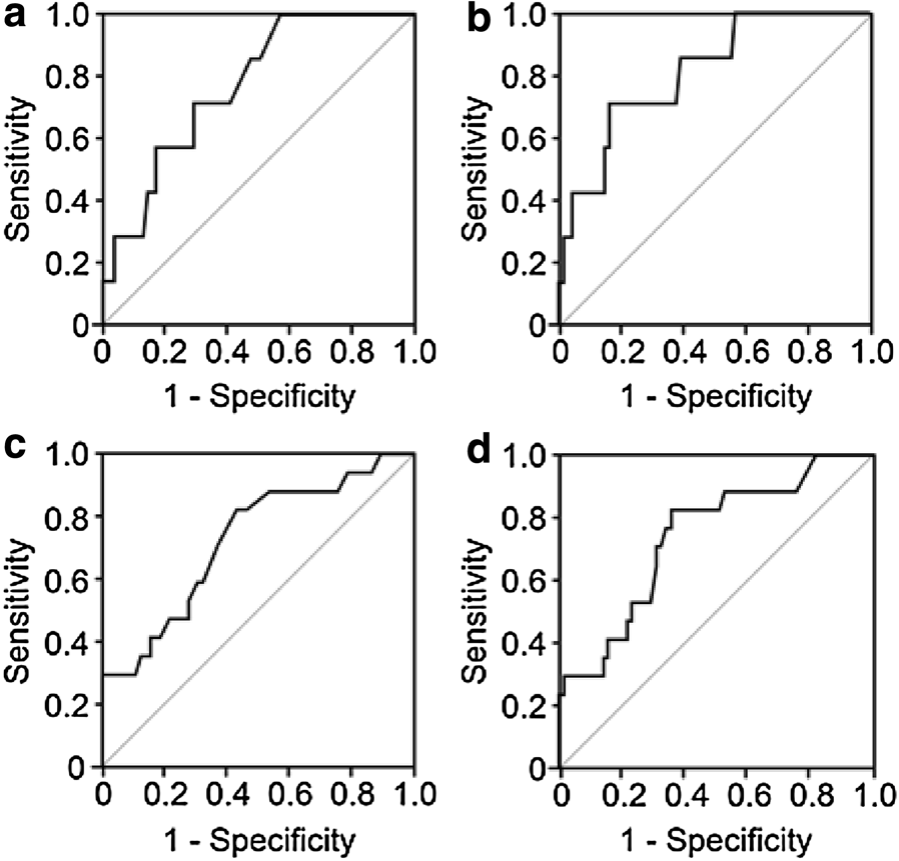
Receiver operating characteristics curve for the FPG and 2 h OGTT used for the detection of diabetes by glucose criteria. **a** Early FPG for DM. **b** Early 2-h OGTT for DM. **c** Early FPG for Pre DM. **d** Early 2-h OGTT for Pre DM

The AUC based on the FPG in the early period for the group with diabetes was 0.77 (95 % CI 0.61–0.92; p = 0.020) and 0.82 (95 % CI 0.66–0.97; p = 0.006) based on the 2-h OGTT (Fig. 1). The AUC based on the FPG in the early period for the group with prediabetes was 0.73 (95 % CI 0.59–0.86 p = 0.004) and 0.74 (95 %CI 0.61–0.87; p = 0.002) based on the 2-h OGTT (Fig. 1). The greatest accuracy for prediabetes was found with the cutoff values of 78 mg/dl (4.3 mmol/L) for FPG and 130 mg/dl (7.2 mmol/L) for the 2-h OGTT (specificity was 63.1 and 64.6 %, sensitivity was 70.6 and 76.5 %, the positive predictive value was 33.3 and 36.1 % and the negative predictive value was 89.1 and 91.3 %, respectively). The greatest accuracy for diabetes was found with the cutoff values of 80 mg/dl (4.4 mmol/L) for the FPG and 150 mg/dl (8.33 mmol/L) for the 2-h OGTT (the specificity was 66.7 and 80 %, the sensitivity was 71.4 % for both, the positive predictive value was 16.7 and 25 % and the negative predictive value was 96.2 and 96.8 %, respectively) (Table 2).

**Table 2:** Optimal early fasting glucose and 2-h OGTT for the determination of dysglycaemia

	Receiver operating curve cutoff value (fasting and postprandial glucose) (mg/dl)	AUC (95 % CI)	Sensitivity (%)	Specificity (%)	Positive predictive value (%)	Negative predictive value (%)
Prediabetes	78	0.73 (0.59–0.86)	70.6	63.1	33.3	89.1
Prediabetes	130	0.74 (0.61–0.87)	76.5	64.6	36.1	91.3
Diabetes	130	0.82 (0.66–0.97)	85.7	60.0	16.7	97.8
Diabetes	150	0.82 (0.66–0.97)	71.4	80.0	25	96.8
Diabetes	78	0.77 (0.61–0.91)	71.4	58.7	13.9	95.7
Diabetes	80	0.77 (0.61–0.91)	71.4	66.7	16.7	96.2

The mean age of women not included in the study (n = 175) were 30.5 (±5.8) years and the mean age of pregnancy at GDM diagnosis were 23.9 (±7.4) weeks, with an average BMI of 28.4 (±5.1) kg/m^2^. In addition, 57.5 % were on insulin and 65.4 % had a family history of diabetes. Comparing both groups (women included and not included in the study), there was no statistically difference in continuous sociodemographic variables. Concerning the categorical variables, the history of previous GDM in the mothers included in the study was significantly higher than in the group of mothers not included (23.4 × 7.9 %, p = 0.008). There was no significant difference in other categorical variables between the two groups (Tables 3, 4).

**Table 3:** Comparison of sociodemographic categorical variables of pregnancy and the newborn among the women included and not included in the study

	Included (%)	Not included (%)	P value
Age (years)			0.085
10–20	0	5.26	
21–30	40.5	43.4	
31–40	50.6	48.7	
41–50	8.9	2.6	
Ethnicity			0.86
Caucasian	45.6	47.1	
Non caucasian	54.4	52.9	
Gestational age at DMG diagnosis (weeks)			0.56
≤20	38.0	33.7	
≥21	62.0	66.3	
Insulin treatment			0.35
Yes	64.6	57.5	
No	35.4	42.5	
Previous history of DMG			0.008
Yes	23.4	7.9	
No	76.6	92.1	
BMI (kg/m^2^)			0.46
<25	34.7	26.9	
25–29.9	31.9	39.7	
30–34.9	25.0	19.2	
35–40	8.3	12.8	
>40	0.0	1.3	
Macrosomia			0.17
Yes	14.9	24.5	
No	85.1	75.5	
Type of delivery			0.14
Vaginal	30.7	18.2	
Caesarean	69.4	81.8	
DM familiar			0.69
Yes	68.4	65.4	
No	31.6	34.6

**Table 4:** Comparison of sociodemographic continuous variables of pregnancy and the newborn among women included (n = 82) and not included (n = 175) in the study

	Included		Not included		P value
	Mean ± SD	Median	Mean ± SD	Median	
Age (years)	32.2 ± 5.8	32	30.5 ± 5.8	31	0.075
Gestational age at diagnosis of GDM (weeks)	23.1 ± 7.4	24	23.9 ± 7.4	25	0.47
Start of insulin (weeks)	28.6 ± 7.1	30	26.9 ± 7.2	28	0.30
Parity (n)	2.3 ± 1.3	2	2.3 ± 1.4	2	0.89
Weight gain until GDM diagnosis (kg)	7.0 ± 5.5	6	8.0 ± 5.6	8.8	0.18
Weight gain until delivery (kg)	10.2 ± 6.3	8.2	11.6 ± 6.0	11.5	0.23
Pregestational BMI (kg/m^2^)	27.7 ± 5.3	27.1	28.4 ± 5.1	27.6	0.41
Birth weight (kg)	3250.3 ± 627.3	3257.5	3409.2 ± 652.0	3400	0.21
Gestational age at delivery (weeks)	37.8 ± 2.9	38	37.8 ± 2.0	38	0.32

We compared the influence of the type of delivery (Vaginal vs Cesarean section) on serum levels of fasting glucose and post-load 75 g, both in collecting 48–72 h as in collecting six weeks, and no statistically significant difference was found.

## Discussion

To our knowledge, this is the first study that evaluate the accuracy and cut off values of OGTT during the early postpartum period in women still hospitalised with previous GDM for determine DM risk. Recently, a study was published and evaluated 58 women with previous GDM who agreed to perform the 75 g OGTT on the second day postpartum. These results were compared with the standard 75 g OGTT 4–12 weeks postpartum. Only 49 of the 98 women presented for routine postpartum OGTT. This study concludes that performing OGTT on the second day is feasible and should be further investigated as an alternative postpartum testing regimen in GDM [36]. In this present study, a 48–72 h OGTT was proven to be a useful tool for identifying women who must perform an OGTT at 6 weeks after delivery. We determined that a FPG of 78 mg/dl (4.3 mmol/L) or a 2-h OGTT of 130 mg/dl (7.2 mmol/L) were the optimal screening cutoff levels for prediabetes, whereas 80 mg/dl (4.4 mmol/L) or 150 mg/dl (8.3 mmol/L), respectively, were the optimal screening cutoff levels for diabetes. Thus, a FPG of 78 mg/dl (4.3 mmol/L) and a 2-h OGTT of 130 mg/dl (7.2 mmol/L) provide the optimal cutoffs to screen for dysglycemia after GDM complicated pregnancies. Additionally, we determined that the 2 h OGTT was more accurate than the FPG.

It is important for screening tests to miss fewer individuals with the target disease, and therefore, screening cutoffs are typically lower compared with diagnostic cutoffs. Differences may also result from interference related to the hormonal changes that occur during this postpartum period [37–39]. A special hormonal milieu is observed between 48–72 h after birth. During pregnancy, the levels of oestrogen and progesterone increase greatly primarily because of placental production [40–43]. Following the removal of the placenta, these hormones fall sharply and reach pre-pregnancy levels on the fifth day postpartum [44]. Levels of cortisol, beta- endorphin, and β-HCG also decline during this period [45]. In women who do not breastfeed, prolactin returns to pre-pregnancy levels by 3 weeks after delivery [46]. These hormones, which are counter-regulatory to insulin, contribute to increased insulin resistance in the early postpartum period. Although the OGTT at 48–72 h did not appear to define the diagnosis of diabetes in this study, it was notable that the OGTT at 48–72 h had a high negative predictive value, with a cutoff FPG <78 mg/dl (4.3 mmol/L) and 2-h OGTT <130 mg/dl (7.2 mmol/L), thereby excluding nearly all individuals with diabetes (but not prediabetes). Moreover, diabetes-screening cutoffs that included an early FPG of 78 mg/dl (4.3 mmol/L) and a 2 h OGTT of 130 mg/dl (7.2 mmol/L) effectively identified higher-risk individuals who required a referral for additional evaluation and management. Using these cutoff points, it was found that only 37 % of women should be advised to have their glucose tolerance assessed 6 weeks after delivery. Thus, more than half of women (63 %) should be reevaluated only after 1–3 years per the ADA guidelines on the frequency of testing [18]. Furthermore, identifying women that should return at 6 weeks eases the overall burden to health-care practitioners and reduces the effort required to contact patients and, if necessary, provide a home visit by a health-care worker, which many studies have recommended [47].

The possibility of performing the screening while women are still in the hospital will most likely enhance the efficacy of detecting women at a higher risk for developing DM and will reinforce the need to return for individuals with a greater risk. Hospital sampling could overcome the barriers that currently prevent women from returning to health-care providers for investigation after 6 weeks. The characteristics of patients associated with higher rates of postpartum screening included older age, nulliparity, and higher income or education [15, 31]. Women who received prenatal care, were treated with insulin during pregnancy, or completed a postpartum visit were also more likely to receive a postpartum diabetes screening [15]. In the present study, 31.9 % of the women attended the postpartum follow-up examination, although all patients received reminders upon completion of the first OGTT. This response rate is similar to the lowest follow-up frequency [15, 31]. The most likely explanation for this result is that women in the studied population predominately belonged to low socioeconomic levels and thus, had lower levels of income and education. Sixty-four percent of patients that were included required insulin treatment, and 56 % of patients had more than 8 years of education. The problem of identifying DM after GDM, which may be of greater concern than the choice of test itself, is the poor postpartum screening rate. Although counselling for the management of chronic disease may be challenging in the postpartum period, individuals sometimes express greater interest in their health during times of illness, and opportunities for early diagnosis should not be lost. During a brief discussion, patients with elevated FPG and/or 2 h OGTT could be encouraged to partner with a provider and maintain long-term care, as well as to attempt lifestyle modifications. The concept of a “teachable moment” has been demonstrated for the case of smoking cessation, in which patients are more likely to quit smoking after health events, such as pregnancy, hospitalisation, and the diagnosis of cancer [39]. Thus, health events represent opportunities for health care providers to educate patients and encourage behaviour modifications [48–50].

The prevalence of diabetes in women after GDM in our study (8.5 %) is consistent with those previously described (5–14 %) [14, 15, 51–53]. The first OGTT identified more prediabetes than diabetes cases compared with the second OGTT (prediabetes 32.9 vs 20.7 %; DM 3.7 vs 8.5 %). This result was most likely the result of the number of patients included. Larger cohorts are necessary to recommend the OGTT at 48–72 h as a test for the early screening of postpartum women with GDM. Differences may also result from interference related to the hormonal changes that occur during this postpartum period [37–39]. A special hormonal milieu is observed between 48–72 h after birth. During pregnancy, the levels of oestrogen and progesterone increase greatly primarily because of placental production [40–43]. Following the removal of the placenta, these hormones fall sharply and reach pre-pregnancy levels on the fifty day postpartum [44]. Levels of cortisol, beta-endorphin, and β-HCG also decline during this period [45]. In women who do not breastfeed, prolactin returns to pre-pregnancy levels by 3 weeks after delivery [46]. These hormones, which are counter-regulatory to insulin, contribute to increased insulin resistance in the early postpartum period.

This study had some limitations. Firstly, patients were included since 2008 and were classified according to two different criteria for GDM diagnosis (Carpenter and Coustan and the IADPSG). Ikenoue et al. suggested that the IADPSG-defined GDM of one abnormal OGTT value indicates a less severe glucose intolerance, but may still signal a risk of requiring insulin when a first-degree family history of diabetes exists [54]. Our participants had a high prevalence of a relative with diabetes (68.4 %). Moreover, women who were discharged prior to 48 h were not included in this study. These patients most likely had a milder case of GDM and lowest prevalence of postpartum DM. Additionally, the sample size is small, therefore the 95 % CIs around the AUROC curves are quite wide.

Indeed, curtailing the rapidly increasing prevalence of early-onset diabetes is a formidable task for health-care practitioners. Additional efforts are necessary to identify these young women as early as possible because they are one of the best groups for which the implementation of a primary prevention strategy is most effective, not only for themselves but also for their offspring and family. Early postpartum screening for DM effectively identifies higher-risk women with previous GDM who require a referral for additional evaluation and management.

## Conclusions

OGTT performed early in postpartum is a useful tool for identifying women with previous GDM who must perform an OGTT 6 weeks after delivery. A diabetes-screening cutoff of FPG of 78 mg/dl (4.3 mmol/L) and a 2-h OGTT of 130 mg/dl (7.2 mmol/L) effectively identified higher-risk individuals who require a referral for additional evaluation and should be further assessed in larger prospective studies.

## Abbreviations

ADA: American Diabetes Association
AUC: area under the curve
BMI: body mass index
DM: diabetes mellitus
FPG: fasting plasma glucose
GDM: gestational diabetes mellitus
IFG: impaired fasting glucose
IGT: impaired glucose tolerance
IADPSG: International Association of Diabetes and Pregnancy Study Groups
OGTT: oral glucose tolerance test
ROC: receiver operating characteristic
NICE: United Kingdom’s National Institute for Health and Clinical Excellence
WHO: World Health Organisation
